# Effects of erythropoietin on depressive symptoms and neurocognitive deficits in depression and bipolar disorder

**DOI:** 10.1186/1745-6215-11-97

**Published:** 2010-10-13

**Authors:** Kamilla W Miskowiak, Maj Vinberg, Catherine J Harmer, Hannelore Ehrenreich, Gitte M Knudsen, Julian Macoveanu, Allan R Hansen, Olaf B Paulson, Hartwig R Siebner, Lars V Kessing

**Affiliations:** 1 Clinic for Affective Disorders, Department of Psychiatry, Copenhagen University Hospital, Rigshospitalet, Copenhagen, Denmark; 2 Department of Psychiatry, University of Oxford, Warneford Hospital, Oxford, UK; 3 Max Planck Institute of Experimental Medicine and DFG Research Center for Molecular Physiology of the Brain (CMPB), Göttingen, Germany; 4 Neurobiology Research Unit (NRU), Copenhagen University Hospital, Rigshospitalet, Copenhagen, Denmark; 5 Danish Research Centre for Magnetic Resonance (DRCMR), Copenhagen University Hospital Hvidovre, Hvidovre, Denmark

## Abstract

**Background:**

Depression and bipolar disorder are associated with reduced neural plasticity and deficits in memory, attention and executive function. Drug treatments for these affective disorders have insufficient clinical effects in a large group and fail to reverse cognitive deficits. There is thus a need for more effective treatments which aid cognitive function. Erythropoietin (Epo) is involved in neuroplasticity and is a candidate for future treatment of affective disorders. The investigators have demonstrated that a single dose of Epo improves cognitive function and reduces neurocognitive processing of negative emotional information in healthy and depressed individuals similar to effects seen with conventional antidepressants. The current study adds to the previous findings by investigating whether repeated Epo administration has antidepressant effects in patients with treatment resistant depression and reverses cognitive impairments in these patients and in patients with bipolar disorder in remission.

**Methods/design:**

The trial has a double-blind, placebo-controlled, parallel-group design. 40 patients with treatment-resistant major depression and 40 patients with bipolar disorder in remission are recruited and randomised to receive weekly infusions of Epo (Eprex; 40,000 IU) or saline (NaCl 0.9%) for 8 weeks. Randomisation is stratified for age and gender. The primary outcome parameters for the two studies are: depression severity measured with the Hamilton Depression Rating Scale 17 items (HDRS-17) [1] in study 1 and, in study 2, verbal memory measured with the Rey Auditory Verbal Learning Test (RAVLT) [2,3]. With inclusion of 40 patients in each study we obtain 86% power to detect clinically relevant differences between intervention and placebo groups on these primary outcomes.

**Trial registration:**

The trial is approved by the Local Ethics Committee: H-C-2008-092, Danish Medicines Agency: 2612-4020, EudraCT: 2008-04857-14, Danish Data Agency: 2008-41-2711 and ClinicalTrials.gov: NCT 00916552.

## Background and objective

### Background

Depression and bipolar disorder are associated with neurodegenerative processes, reduced neuroplasticity and neuropsychological dysfunction, which often persist even after clinical remission. Current pharmacological treatment strategies have several limitations including a significant treatment-onset-response delay, only partial or no response in a large group of patients and limited effects on their cognitive deficits, which often persist into periods of remission [4-9]. These enduring cognitive impairments affect patients' psychosocial and occupational function, quality of life and prognosis [7,8,10,11]. Better treatment options are thus required to improve the onset of efficacy, address patients who are treatment resistant and remediate neurocognitive deficits. Converging evidence suggests that neural atrophy and reduced neuroplasticity are involved in depression and bipolar disorder, while restoration of synaptic plasticity may be an important mechanism of chronic antidepressant and mood stabilising drug treatment [12-14]. This hypothesis is supported by evidence that increased hippocampal brain-derived neurotrophic factor (BDNF) is necessary for the behavioural effects of antidepressant treatment [15] and that infusion of BDNF into the hippocampus mimics the behavioural effects of antidepressants in rodents [16]. These effects and the role of BDNF in learning and memory suggest that compounds, which induce rapid and prolonged upregulation of neuroplasticity, may reduce the latency to antidepressant effects and alleviate neurocognitive dysfunction.

Erythropoietin (Epo) has interesting properties, which make it a candidate for investigation as a novel therapeutic agent in neuropsychiatric diseases. It is used for the treatment of anaemia but has received renewed attention because of its direct neurobiological actions mediated through a non-haematopoietic Epo receptor system in the brain [17]. Systemically administered Epo crosses the blood-brain barrier (BBB) and has neuroprotective and neurotrophic effects in animal models of acute brain damage and chronic neurodegenerative conditions [18,19]. In humans, repeated Epo administration improves neuropsychological function in patients with schizophrenia [20] and multiple sclerosis [21]. Several complex biochemical pathways mediate these actions, including rapid up-regulation of neuroplasticity mechanisms including BDNF and neurogenesis [22,23], and anti-inflammatory actions [24,25].

Functional magnetic resonance imaging (fMRI) evidence highlights the functional neuroanatomy of emotional processing and working memory as potential neurobiological markers for depression [26,27]. Antidepressant drugs with different neurochemical actions show similar effects in such human biomarker models relevant to depression and antidepressant effects. In particular, serotonergic and noradrenergic antidepressants increase the neural and cognitive processing of positive emotional information and down-regulate the processing of negative threat-relevant information after acute and 7 days administration and in the absence of changes in mood in healthy volunteers [28,29] and in depressed patients [30]. Such actions contrast with heightened recognition and neural responses to threatening information in depression [31,32] and may be an important mechanism of antidepressant drug treatment. We have recently reported cognitive and neural effects of a single dose of Epo (40,000 IU; i.v.) in human models of antidepressant drug action independent of any haematological effects in healthy volunteers [33-36]. In particular, Epo enhanced memory-relevant hippocampal engagement during picture retrieval 1 week post administration [34] consistent with increased hippocampal BDNF and plasticity [37,38]. Epo also modulated fronto-parietal activation during spatial working memory (WM) and improved verbal fluency performance at this time after administration [35]. Finally, Epo reduced parietal-occipital activation during presentation of fearful facial expressions and the behavioural recognition of this emotion [33] consistent with decreased attentional processing and perception of threat-relevant stimuli 7 days post administration similar to effects seen with conventional antidepressant drug administration to healthy volunteers [28,29]. Notably, we demonstrated a similar reduction in the neural and cognitive response to negative threat-relevant picture stimuli following a single dose of Epo vs. placebo to acutely depressed patients [39]. Together with its known effects on neuroplasticity in animal models, these findings highlight Epo as a new candidate treatment for affective disorders.

### Objective

The aim of the study is to investigate whether these early effects of Epo translate into improvement of mood and neurocognitive function with repeated administration in patients with treatment-resistant depression or bipolar disorder in remission and to elucidate the underlying biomarkers of potential treatment effects. Beneficial effects on these measures would highlight Epo as a candidate for adjunct treatment for affective disorders to attenuate mood symptoms and aid cognitive function in these patients groups.

## Methods

### Study design

The present trial has a double-blind, placebo-controlled, parallel-group design in which participants are randomised to receive weekly infusions of either Epo (Eprex; 40,000 IU; Janssen-Cilag) or saline over 8 weeks. Patients complete a broad neurocognitive test battery assessing memory, executive function and attention at baseline, after completion of treatment (week 9) and at follow-up (week 14). Mood, symptoms and quality of life are measured at baseline and weeks 5, 9 and 14 using a variety of rating scales including the Hamilton Depression Rating Scale 17 items (HDRS-17) [1], Young Mania Rating Scale (YMRS) [40], Beck Depression Inventory (BDI) [41], Massachusetts General Hospital Cognitive and Physical Functioning Questionnaire (CPFQ) [42], and the EuroQol visual analogue scale (VAS) for quality of life (EQ-5D) [43]. Patients undergo an fMRI scan at baseline and week 14. Further, plasma and whole blood BDNF and inflammatory markers will be investigated with blood tests at baseline and weeks 5, 9 and 14. Finally, we will assess metabolic parameters with blood tests at baseline and weeks 5, 9 and 14, and body fat mass by Dual energy X-ray absorptiometry (DXA) scanning at baseline and week 9. For an overview of investigations and their timing see table 1.

**Table 1:** Overview of investigations

	Interventions/Assessment domains	Tests/Evaluations	Screening	Baseline & treatment start	Active treatment phase	Treatment-free follow-up period
			
				Week 1	(Week 2-8)	(Week 9-14)
**Treatment**	Investigational drug	EPO (40,000 IU)/Placebo	-	1×	7× (weekly)	-

**Efficacy**	Neurocognitive functions	Neuropsychological testing (120 min)	-	1×	-	2× (week 9,14)
		
		MRI, fMRI and cognitive tests	1×	-	-	1× (week 14)
	
	Psychopathology	Clinical Rating	-	1×	1× (week 5)	2× (week 9,14)
		
		Questionnaires	-	1×	1× (week 5)	2× (week 9,14)
	
	Quality of life questionnaires		-	1×	1× (week 5)	2× (week 9,14)
	
	DXA scan		-	1×	-	1× (week 9)

**Safety**	Physical exam		1×	1×	7× (weekly)	3× (week 9,10,14)
	
	Vital signs		1×	1×	7× (weekly)	3× (week 9,10,14)
	
	Urine pregnancy test		1×	1×	3× (week 3,5,7)	-
	
	ECG		1×	-	2× (week 3,5)	-
	
	Adverse event monitoring		-	* Continuously until week 10*		
	
	Blood analysis	Haematology	1×	1×	7× (weekly)	3× (week 9,10,14)
		
		Blood chemistry	1×	1×	7× (weekly)	3× (week 9,10,14)
		
		BDNF, inflammatory and metabolic markers	-	1×	1× (week 5)	2× (week 9,14)

### Participants and screening

Patients with treatment-resistant major depression (study 1) and patients with bipolar disorder in full or partial remission but with cognitive problems (study 2) will be recruited through Clinic for Affective Disorders, Department of Psychiatry, Copenhagen University Hospital, Rigshospitalet. The centre receives patients with treatment-resistant major depression or bipolar disorder from the whole Capital Region. Patients will be screened with Schedules for Clinical Assessment in Neuropsychiatry (SCAN) [44] to confirm diagnosis.

Inclusion Criteria are a diagnosis of either treatment-resistant major depression (defined as failure to respond to at least two different types of antidepressants given in sufficient doses over sufficient time) in moderate to severe degree (HDRS score ≥ 17), or bipolar disorder in full or partial remission (HDRS ≤ 14 and YMRS ≤ 14) and subjective experience of moderate to severe cognitive problems on the CPFQ (score ≥ 4 on ≥ 2 domains), age between 18-65 years and unchanged antidepressant or mood stabilising treatment for minimum 2 weeks prior to inclusion in the study.

Exclusion criteria are any significant medical conditions (incl. diabetes, renal failure, epilepsy, untreated hypertension, present or past malignancies and thrombosis), smoking, hypertension, overweight (BMI > 30) or a body weight < 45 or > 95 kg, schizophrenia, present alcohol or substance misuse, acute suicidal risk, present or previous suicide attempts in the past 2 years, pregnancy or breast feeding, contraceptive medication, or a first-degree family history of thromboembolic events or seizure disorders.

A pregnancy test will be performed on female patients in their fertile age before and every second week during the study. Blood screening and physical examinations will be performed at baseline and every week during the treatment period as well as 1, 2 and 6 weeks after treatment cessation to monitor patients' level of red blood cells and ensure patient safety. Written informed consent will be obtained from all patients before their inclusion in the study and letters sent to their general practitioners to avoid any potential medical complications.

### Randomisation

Block randomisation for the two studies has been performed by Pharma Consulting Group AB http://www.pharmaconsultinggroup.com. Pharma Consulting Group has created randomisation lists for each study and emergency envelopes for each patient ID number. Treatment groups are stratified for age (< or ≥ 35 years) and gender. Upon inclusion of participants, the date of birth, gender, diagnosis, and to which of the above strata the participant belongs are recorded. Numbering within each stratum is consecutive and study identification numbers are recorded in the patients' individual case report forms (CRFs).

### Blinding

Study personnel involved in the evaluation of outcomes and Epo/placebo infusions and study participants are blinded to study medication allocation. Blinding is maintained throughout the study, data management, outcome assessment and data analysis. This is possible because Epo is a clear colorless liquid similar to saline and because safety monitoring and bloodlettings are performed by a medic who is not involved in the evaluation of outcome measures or drug administration.

### Outcome assessments

The primary outcome for treatment-resistant depressed patients (study 1) is depression severity as measured with the HDRS-17 [1] at week 9. Global Assessment of Function (GAF) scores at week 9 will be reported in addition to the primary outcome. The secondary outcome is remission defined as HDRS score ≤ 8 at week 9. Confidence intervals will be reported in relation to the proportions in remission. The tertiary outcomes are memory and executive functions, measured with the Rey Auditory Verbal Learning Test (RAVLT) [2,3] and Rapid Visual Processing (RVIP) test from the Cambridge Neuropsychological Test Automated Battery (CANTAB), respectively, facial expression recognition measured with a facial expression recognition test, as well as self-ratings of cognitive difficulties on the CPFQ, depressive symptoms and life quality at week 9.

For bipolar patients in remission (study 2) the primary outcome is memory performance measured with the RAVLT at week 9. The secondary outcome is sustained attention measured with the RVIP test and recognition of emotional facial expressions assessed with a facial expression recognition test at week 9. Finally, the tertiary outcomes are ratings of mood, symptoms, quality of life and cognitive function at week 9.

Clinical mood ratings and patients' self-ratings will be performed at baseline, and weeks 5, 9 and 14 to assess the speed and duration of potential effects on these measures. Neuropsychological testing is performed at baseline, and weeks 9 and 14 to assess effects of Epo vs. placebo treatment and their duration. For overview see table 1.

### Hypotheses

For study 1, it is hypothesised that Epo vs. placebo treatment will: 1) reduce HDRS-17 scores 2) increase remission rates measured with the HDRS-17 3) improve memory and executive function, reduce the perception of negative facial expressions, and improve symptoms and quality of life.

Study 2 is designed to test the hypothesis that Epo vs. placebo treatment will: 1) improve memory 2) facilitate sustained attention and reduce the perception of negative facial expressions measured, and 3) improve mood, symptoms and life quality.

### Further outcome assessments: biomarkers of potential clinical effects

fMRI is performed before and after treatment to investigate the neurophysiological underpinnings for effects of repeated Epo administration on cognitive function and mood. The second fMRI scan is scheduled 6 weeks after treatment cessation (week 14) because hematocrit levels in the two groups should be comparable at this time point [20] and thus not confound data interpretation. We will investigate whether potential memory improvement after Epo vs. placebo administration is associated with increased hippocampal engagement during picture encoding consistent with increased hippocampal BDNF and plasticity [34,38]. The neural correlates of potential improvements in executive function with Epo vs. placebo treatment will be investigated with a spatial N-back working memory test. Finally, we will investigate whether potential antidepressant effects of Epo are accompanied by reduced cognitive and neural response to fearful facial expressions in a face-processing test similar to effects seen with existing antidepressants [32]. This would point to the modulation of emotional bias as a common mechanistically important effect of compounds with diverse neurochemical signalling mechanisms. Structural T1-weighted MRI and diffusion weighted imaging (DWI) at these time points will investigate whether Epo-treatment induces structural changes in the hippocampus and influences functional brain connectivity, respectively. Arterial spin labelling (ASL) will investigate whether Epo introduces enduring changes in global and regional cerebral blood flow (after the return of haematocrit levels to baseline).

It is known that Epo increase hippocampal BDNF and nerve growth factor (NGF) and has anti-inflammatory actions, but it is unclear whether these actions are mechanistically important for potential antidepressant effects of Epo. We will therefore investigate the effect of Epo on plasma and whole blood BDNF, NGF and inflammatory markers incl. interleukin-6 (IL-6) and C-reactive protein (CRP). Given diurnal variation in peripheral BDNF, blood samples are collected at approximately the same time for all patients. For overview of timing see table 1.

Preclinical research suggests that Epo protects against diet-induced obesity and has beneficial effects on metabolic parameters [45], which may play a role in the treatment of affective disorder [46]. The study therefore investigates the effects of Epo on metabolic parameters (fasting glucose, fasting insulin, HgbA1c, HDL-cholesterol, LDL-cholesterol and TAG) in these patients through blood sampling before and during Epo vs. placebo treatment. DXA-scanning (Lunar Prodigy, GE Medical Systems Wisconsin, USA, version 8.8) is performed to assess whether Epo affects the total lipid- and lipid-free lean mass in the body. For overview of timing see table 1.

### Sample size and power calculation

Sample size and statistical power for the two studies were calculated using nQuery Advisor 5.0 software. The primary outcome in study 1 is depression severity reflected by HDRS-17 after Epo vs. placebo treatment. The clinically relevant difference is defined as minimum 3 scores on this scale. The mean standard deviation in the two treatment groups is estimated to be 3 HDRS-17 scores and the mean scores to be 18 and 21 after Epo and placebo treatment, respectively. With these assumptions a sample of n = 40 patients (n = 20 per drug group) has a statistical power of 86% to detect a clinically relevant difference in HDRS-17 scores between the two treatment groups at an alpha level of 5% (2-sided test).

The primary outcome in study 2 is verbal memory, reflected by the RAVLT total score after Epo vs. placebo treatment. A recent study has shown that the mean RAVLT total score for patients with bipolar disorder in remission is 52.0 (while the mean score for healthy age-matched controls was 60.7) out of a maximum of 75 [47]. We estimate that a clinically relevant difference between drug groups in the change in RAVLT scores is at least 4 points (half-way to normal function). The mean standard deviation in RAVLT scores in the two treatment groups is estimated to be 4 points and the mean scores to be 56 and 52 after Epo and placebo treatment, respectively. With these assumptions a sample of n = 40 patients (n = 20 per drug group) has a statistical power of 86% to detect a clinically relevant difference in RAVLT total scores between the two treatment groups at an alpha level of 5% (2-sided test).

### Psychological tests

The key aspects of cognitive function affected in mood disorder, executive function, attention, memory and emotional processing, are assessed using an extensive battery of neurocognitive tests. The test battery includes following manual tests: RAVLT, Verbal and Semantic Fluency Tests, WAIS-III Letter Number Sequencing, Trail Making A and B, the Repeatable Battery for the Assessment of Neuropsychological Status (RBANS) digit span and coding tests, and the following computerised tests: Simple Reaction Time, Rapid Visual Information Processing, Delayed Match to Sample and Spatial Working Memory. Tests used for fMRI scanning are an N-back working memory task, a picture encoding task modified from Hariri et al [38], an emotional face processing task using fearful and happy Nimstim picture stimuli http://www.macbrain.org/resources.htm and a facial expression recognition task using Ekman and Friesen morphed stimuli sets [48].

### Equipment

The computerised neuropsychological test battery CANTAB (Cambridge Cognition Ltd.) are employed for a precise estimation of drug effects on accuracy and reaction times during the above mentioned tasks probing executive function, attention and memory. The effects of Epo on the neural basis of executive function, memory and emotion processing as well as potential structural changes are investigated using the in vivo non-invasive techniques of fMRI, structural T1 weighted MRI, DWI, and ASL-based measurements of cerebral blood flow. Imaging data will be collected using a Siemens Trio scanner operating at 3.0 T, at the Danish Research Centre for Magnetic Resonance (DRCMR), Copenhagen University Hospital, Hvidovre. fMRI data will pre-processed and analysed using FEAT (FMRIB Expert Analysis Tool) version 5.43, part of FSL (FMRIB Software Library version 3.3) http://www.fmrib.ox.ac.uk/fsl. Structural T1-weighted MRI data is analysed with FAST (FMRIB's Automated Segmentation Tool), FIRST (FMRIB's Integrated Registration and Segmentation Tool) http://www.fmrib.ox.ac.uk/fsl and FreeSurfer http://surfer.nmr.mgh.harvard.edu/. DWI data is analysed with FDT (FMRIB's Diffusion Toolbox; http://www.fmrib.ox.ac.uk/fsl/fdt/). Finally analysis of ASL data is performed with SPM software http://www.fil.ion.ucl.ac.uk/spm/software/spm8/ and FABBER http://www.fmrib.ox.ac.uk/fsl/fabber.

### Statistical analyses

Data will be collected until dropout from all randomised patients and analysed using repeated measures analysis of variance (ANOVA). Significant interactions were analysed further with simple main effect analyses. In the case of missing or invalid data, we will perform Per Protocol (PP) analyses for complete data sets and Intention to Treat (ITT) analysis. In the case of disparity between the results of these analyses, the outcome of the ITT analysis will be regarded as the result of the study. The statistical threshold for which results are considered significant if p ≤ 0.05 (2-tailed). Statistical analyses will be performed using the Statistical Package for Social Sciences (SPSS).

### Data management

Data for each participant is kept in a Case Record File (CRF), which fulfil the Danish law for medical doctors' obligation to keep patients records. The study personnel involved in the evaluation of clinical outcomes are blinded to the treatment until the data analysis is completed. Blood test results and lists of potential adverse effects are therefore kept separately in a locked safe to which only medical doctors responsible for patient safety and the person involved in the blinding of the study medication have access.

### Safety

#### Safety profile of Epo

Epo is widely used in the treatment of anaemia and generally has a good safety profile. Some potential acute side effects are transient flu-like symptoms, rash at the injection site and headache, which are normally reported as mild and disappear within a few hours. However, long-term Epo-treatment can induce epileptic seizures in medically ill patients with chronic renal failure and past history of seizures [49]. A very rare adverse effect of Epo treatment is a condition where the patient develops neutralising antibodies against Epo resulting in pure red cell aplasia (PRCA). This problem has only been observed in patients treated with subcutaneous injections and was solved in 2003 when the manufacturer changed the preparation, packaging and transport of Epo [50]. Today, Epo-antibody formation is thus an extremely rare condition observed with long-term subcutaneous Epo-treatment of patients with chronic renal failure [51].

Meta-analysis of 5243 renal failure patients in long-term Epo treatment showed a slight increase in mortality (17%; 95% C.I. 1-35%) potentially because of thrombosis at patients' arterial dialysis access site and insufficient treatment of hypertension [52]. Recently, a study of 522 stroke patients treated with Epo vs. placebo also revealed greater mortality in the Epo group, possibly because of interaction with recombinant tissue plasminogen activator (rtPA) used for thrombolysis as well as an unusual low death rate in the placebo group [53].

Weekly high-dose (40,000/48,000 IE) Epo administration to patients with neuropsychiatric disorder increases haematocrit after 5 weeks [20,21] calling for bloodlettings in about 40% of patients when values exceed 50% and 48% for men and women, respectively. However, continued Epo-treatment in the absence of any iron substitution was well tolerated and not associated with further haematocrit increase after 8 weeks of treatment [20,21].

#### Safety precautions

Given the above safety concerns and increased mortality in certain patient groups, the study inclusion and exclusion criteria (see 'Patients and screening') are strictly adhered to and patients' safety monitored closely throughout the study. Given the very low incidence of PRCA, our use of intravenous (vs. subcutaneous) administration and exclusion of patients with renal failure, we believe that the risk of PRCA in the current study is negligible. Nevertheless, reticulocyte counts as first indicator of PRCA will be carefully monitored (see below).

Patients are informed of all potential adverse effects before their inclusion in the study, instructed that iron supplements are strictly prohibited (since they would boost haematocrit levels) and advised to immediately contact their local emergency department if (1) they experience any potential symptoms of thrombosis or (2) their physician suspects PRCA. They are given a pocketsize plastic card with instruction about whom to contact in the event of these symptoms, their contact details, and important information to the medical doctor at the local emergency department.

It is ensured the temperature of the Epo is kept at 2-8°C at all times during transport and storage to avoid damage of the compound and potential associated adverse effects. To minimise the risk of acute allergic reactions, Epo/placebo is dissolved in 100 ml saline and administered as intravenous infusions over 15 minutes. After this, patients' well-being is monitored for at least 30 minutes by a nurse at the clinic in the case of potential transient side effects of Epo like flu symptoms, rash at the infusion site or head ache.

#### Safety parameters and monitoring

Patient safety is monitored with medical examinations, blood pressure measurements, blood tests, ECG and other safety measures at baseline and throughout the study until 2 weeks after cessation of Epo/placebo treatment. For an overview of the safety tests, their frequency and timing see table 1.

If blood tests show significantly increased haematocrit (>50% for men and >48% for women) at two consecutive measurements within a week, bloodletting(s) (450 ml each) will be performed weakly with no cessation of treatment until haematocrit values are normalised. In the case of significant increase in thrombocytes (> 400 billion/L) or drop in reticulocytes (< 1‰) 2 repeated controls will be performed in the following week. If these values remain abnormal, the patient is taken out of the study and monitored with weekly medical examinations and blood tests until the values are normalised or is hospitalised for observation.

#### Procedures for acting on serious adverse effects

Procedures for breaking the randomisation code are established for the case of severe adverse reactions potentially related to the drug intervention. These will be followed when knowledge of the patient's drug treatment will have implications of the treatment of the observed adverse effects. It is the decision of the principal investigator or the medic responsible for safety monitoring to break the emergency envelope for the affected participant.

## Discussion

The present EPO study is the first clinical trial investigating whether repeated administration of Epo is able to improve mood and reverse neurocognitive dysfunction in patients with treatment resistant depression or bipolar illness in remission. In light of the hypothesis that neural plasticity and neurogenesis play a prominent role in the pathophysiology of affective disorders, cognitive function and clinical response to drug treatment, Epo is a compound worth to be considered for future add-on treatment strategies. It is hypothesised that Epo treatment will have antidepressant effects in patients with treatment resistant depression and alleviate the enduring neurocognitive dysfunction in patients with bipolar disorder in remission.

### Current trial status

Patient enrolment started in September 2009 and is ongoing until September 2013. Status in October 2010 is that 21 participants have been screened, enrolled and randomised. One patient has had to drop out after week 5 because of abnormal laboratory results. So far, data sets including all clinical variables and biomarkers are complete for 18 participants.

### Ethical considerations

Better treatment options are required to improve the onset of efficacy, address the significant proportion of patients who are treatment resistant and reverse neurocognitive deficits in patients with affective disorders. Compounds, which induce rapid and prolonged upregulation of neuroplasticity may reduce the latency to antidepressant effects and alleviate neurocognitive dysfunction. Epo has such neuroregenerative properties, which make it a candidate for investigation as a novel therapeutic agent. We have recently demonstrated a beneficial effect of Epo on neurocognitive function and mood in healthy volunteers and antidepressant-like effects in acutely depressed patients. However, it is unclear whether repeated Epo treatment has antidepressant effects and improves neurocognitive function in patients with affective disorders. The present study will thus increase our knowledge about the role of neuroplasticity in affective disorders and perhaps contribute to development of new, better treatment strategies.

Participants in the present study are patients with treatment-resistant depression (study 1) and patients with bipolar disorder in full or partial remission who suffer from persistent cognitive difficulties (study 2). For both patient groups there are a number of benefits related to taking part in the study. For treatment-resistant depression, defined as lack of effect of medication from minimum 2 different classes of antidepressants given in sufficient doses over sufficient time [54,55], there is no scientific evidence for effect of additional pharmacological treatment. Further, cognitive function remains impaired in many patients with bipolar disorder even after clinical remission. If the study reveals positive effects of Epo on mood and cognitive function, this would highlight Epo as a new potential treatment for these patient groups in the future. All patients have close contact to a medical doctor, a research nurse and a psychologist for the duration of the study, which has shown beneficial effects in previous studies [56]. Finally, patients are remunerated for their time and transport expenses related to taking part in the study.

There are also some risks and inconveniences for patients in the study. Although they will maintain their current antidepressant or mood stabilising treatment, they are requested to not make any changes in their medication for the duration of the study. This may give rise to ethical considerations for the acutely depressed patients in study 1. Nevertheless, these patients are characterised by treatment-resistance to currently available antidepressant medication and it is unclear which treatment would help this group. Therefore, keeping patients' treatment constant for the duration of the Epo/placebo treatment does not impose any significant ethical dilemmas. Finally, there are some health risks associated with Epo treatment as previously described. However, we consider these to be negligible in light of safety data from previous Epo studies and the described exclusion criteria, safety precautions and close safety monitoring performed throughout the study.

Given the above ethical considerations, we believe that participants' risks and inconveniences are minimal and outweighed by their benefits and by the potential implications of the trial for future management of mood symptoms and cognitive functioning in these patient groups.

### Limitations

A major limitation of the study design is the implementation of many exclusion criteria (see 'Patients and screening'), which serves to maximise participant safety. This limits the generalizability of the findings to clinical practice. Specifically, our sample may not be particularly representative for the full range of physical and psychiatric comorbidity, response to treatment and functioning of patients seen in the clinic. Nevertheless, this limitation is considered inevitable since patient safety is of principal importance.

From a clinical perspective there are two potential complications of long-term application of Epo to patients with affective disorders. The first problem is the increases in the red cell mass observed with repeated administration leading to increased risk of hypertension and blood clotting [57]. Future studies may wish to assess whether such unwanted haematopoietic activities can be avoided with infrequent Epo administration while maintaining the neuroprotective effects. Another solution could be the use of modified Epo molecules such as carbamylated Epo (CEPO) and asialoerythropoietin (asialoEPO) which have been engineered to have neuroprotective but not haematopoietic actions [58-60]. In the absence of haematological activities, CEPO and asialoEPO exhibit a broad spectrum of neuroprotective activities in models of cerebral ischemia, spinal cord injury and sciatic nerve crush [58,61-63]. These Epo derivatives may thus be promising candidates for long-term treatment of affective disorders.

The second major limitation of Epo as a clinical treatment of affective disorders is the availability of only intravenous or subcutaneous administration routes, which by many is experienced as unpleasant and increases the costs of EPO treatment. However, alternative administration routes with easier application and lower costs are currently being explored. In particular, intranasal administration of small doses of Epo has recently been found to penetrate the BBB and attenuate acute cerebral ischemic injuries in rats [64]. This suggests that intranasal administration of Epo could become a simple and non-invasive method to treat acute brain trauma and neuropsychiatric conditions in the future. Another clinical benefit of intranasal application is the necessity of significantly smaller doses of Epo for neuroprotection, which may reduce the potentially systemic side effects resulting from erythropoietic activity [64]. Assessment of the safety of intranasal Epo administration in man is thus an important next step before assessment of the neurocognitive effects of Epo with this route of administration in man.

### Perspectives

If the present trial reveals beneficial effects of Epo on mood and neurocognitive function in patients with treatment resistant depression and patients with remitted bipolar disorder, this would highlight Epo and its non-haematopoietic derivatives as candidate compounds for future 'cognitive enhancement' treatment strategies for affective disorders. This could have significant implications for patient health and economical burden for the society in the future.

## Competing interests

KM, JM, HN, AH, GMK, OP and HS declare to have no competing interest.

MV has been a speaker for Eli Lilly, Wyeth, AstraZeneca, Janssen-Cilag and Pfizer.

LVK has been a consultant for Bristol-Myers Squibb, Eli Lilly, Lundbeck, AstraZeneca, Pfizer, Wyeth, Janssen-Cilag and Servier.

CH has received grant support from the Medical Research Council, University of Oxford, Merck, Sharpe, and Dohme. Has acted as a consultant for Lundbeck, Merck, Sharpe, Dohme, Servier, GSK and P1Vital. Holds shares in P1vital.

HE holds/has submitted user patents for EPO in stroke, schizophrenia, and multiple sclerosis.

## Authors' contributions

KM and LVK have conceived of the study. KM, LVK and MV have written up the study protocol. KM has acquired the necessary funding for the study. KM, LVK, MV, GMK, OP, HS, CH and HE have been involved in revising and optimising the study protocol. LVK is coordinating investigator and sponsor for the study. KM is responsible for communication with the governing institutions (Danish Medicine Agency, the local ethics committee, Danish data protection agency and the Good Clinical Practice (GCP) unit as well as all diagnostic interviews and clinical ratings. MV is responsible for inclusion of patients in the study and safety monitoring. All authors have read and approved of the final version of the manuscript.

## References

[B1] Hamilton M A Rating Scale for Depression Journal of Neurology Neurosurgery and Psychiatry 1960 23 56 62 10.1136/jnnp.23.1.56 PMC495331 14399272

[B2] Rey A Psychological examination of traumatic encephalopathy Archieves de Psychologic 1941 28 286 340 Ref Type: Generic

[B3] Rey A L'examen clinique en psychologie. Paris 1964

[B4] Clark L Goodwin GM State- and trait-related deficits in sustained attention in bipolar disorder Eur Arch Psychiatry Clin Neurosci 2004 254 61 68 10.1007/s00406-004-0460-y 15146334

[B5] Dittmann S Hennig-Fast K Gerber S Seemuller F Riedel M Severus W Cognitive functioning in euthymic bipolar I and bipolar II patients Bipolar Disorders 2008 10 877 887 10.1111/j.1399-5618.2008.00640.x 19594503

[B6] Kessing LV Cognitive impairment in the euthymic phase of affective disorder Psychol Med 1998 28 1027 1038 10.1017/S0033291798006862 9794010

[B7] Martinez-Aran A Vieta E Colom F Reinares M Benabarre A Gasto C Cognitive dysfunctions in bipolar disorder: evidence of neuropsychological disturbances Psychother Psychosom 2000 69 2 18 10.1159/000012361 10601830

[B8] Martinez-Aran A Vieta E Colom F Torrent C Sanchez-Moreno J Reinares M Cognitive impairment in euthymic bioplar patients: implications for clinical and functional outcome Bipolar Disorders 2004 6 224 232 10.1111/j.1399-5618.2004.00111.x 15117401

[B9] Zubieta JK Huguelet P O'Neil RL Giordani BJ Cognitive function in euthymic Bipolar I Disorder Psychiatry Research 2001 102 9 20 10.1016/S0165-1781(01)00242-6 11368835

[B10] Chamberlain SR Sahakian BJ Cognition in mania and depression: psychological models and clinical implications Curr Psychiatry Rep 2004 6 451 458 10.1007/s11920-004-0010-3 15538994

[B11] Wingo AP Harvey PD Baldessarini RJ Neurocognitive impairment in bipolar disorder patients: functional implications Bipolar Disorders 2009 11 113 125 10.1111/j.1399-5618.2009.00665.x 19267694

[B12] Berton O Nestler EJ New approaches to antidepressant drug discovery: beyond monoamines Nat Rev Neurosci 2006 7 137 151 10.1038/nrn1846 16429123

[B13] Frey BN Andreazza AC Nery FG Martins MR Quevedo J Soares JC The role of hippocampus in the pathophysiology of bipolar disorder Behav Pharmacol 2007 18 419 430 10.1097/FBP.0b013e3282df3cde 17762510

[B14] Manji HK Moore GJ Rajkowska G Chen G Neuroplasticity and cellular resilience in mood disorders Mol Psychiatry 2000 5 578 593 10.1038/sj.mp.4000811 11126389

[B15] Santarelli L Saxe M Gross C Surget A Battaglia F Dulawa S Requirement of hippocampal neurogenesis for the behavioral effects of antidepressants Science 2003 301 805 809 10.1126/science.1083328 12907793

[B16] Shirayama Y Chen AC Nakagawa S Russell DS Duman RS Brain-derived neurotrophic factor produces antidepressant effects in behavioral models of depression J Neurosci 2002 22 3251 3261 11943826 10.1523/JNEUROSCI.22-08-03251.2002 PMC6757539

[B17] Brines M Cerami A Emerging biological roles for erythropoietin in the nervous system Nature Reviews Neuroscience 2005 6 484 494 10.1038/nrn1687 15928718

[B18] Brines ML Ghezzi P Keenan S Agnello D de Lanerolle NC Cerami C Erythropoietin crosses the blood-brain barrier to protect against experimental brain injury Proc Natl Acad Sci USA 2000 97 10526 10531 10.1073/pnas.97.19.10526 10984541 PMC27058

[B19] Sattler MB Merkler D Maier K Stadelmann C Ehrenreich H Bahr M Neuroprotective effects and intracellular signaling pathways of erythropoietin in a rat model of multiple sclerosis Cell Death Differ 2004 11 Suppl 2 S181 S192 10.1038/sj.cdd.4401504 15459752

[B20] Ehrenreich H Hinze-Selch D Stawicki S Aust C Knolle-Veentjer S Wilms S Improvement of cognitive functions in chronic schizophrenic patients by recombinant human erythropoietin Molecular Psychiatry 2007 12 206 220 10.1038/sj.mp.4001907 17033631

[B21] Ehrenreich H Fischer B Norra C Schellenberger F Stender N Stiefel M Exploring recombinant human erythropoietin in chronic progressive multiple sclerosis Brain 2007 130 2577 2588 10.1093/brain/awm203 17728357

[B22] Viviani B Bartesaghi S Corsini E Villa P Ghezzi P Garau A Erythropoietin protects primary hippocampal neurons increasing the expression of brain-derived neurotrophic factor J Neurochem 2005 93 412 421 10.1111/j.1471-4159.2005.03033.x 15816864

[B23] Wang L Zhang Z Wang Y Zhang R Chopp M Treatment of stroke with erythropoietin enhances neurogenesis and angiogenesis and improves neurological function in rats Stroke 2004 35 1732 1737 10.1161/01.STR.0000132196.49028.a4 15178821

[B24] Villa P Bigini P Mennini T Agnello D Laragione T Cagnotto A Erythropoietin selectively attenuates cytokine production and inflammation in cerebral ischemia by targeting neuronal apoptosis J Exp Med 2003 198 971 975 10.1084/jem.20021067 12975460 PMC2194205

[B25] Yatsiv I Grigoriadis N Simeonidou C Stahel PF Schmidt OI Alexandrovitch AG Erythropoietin is neuroprotective, improves functional recovery, and reduces neuronal apoptosis and inflammation in a rodent model of experimental closed head injury FASEB J 2005 19 1701 1703 16099948 10.1096/fj.05-3907fje

[B26] Fu CHY Mourao-Miranda J Costafrecla SG Khanna A Marquand AF Williams SCR Pattern classification of sad facial processing: Toward the development of neurobiological markers in depression Biological Psychiatry 2008 63 656 662 10.1016/j.biopsych.2007.08.020 17949689

[B27] Marquand AF Mourao-Miranda J Brammer MJ Cleare AJ Fu CHY Neuroanatomy of verbal working memory as a diagnostic biomarker for depression Neuroreport 2008 19 1507 1511 10.1097/WNR.0b013e328310425e 18797307

[B28] Harmer CJ Shelley NC Cowen PJ Goodwin GM Increased positive versus negative affective perception and memory in healthy volunteers following selective serotonin and norepinephrine reuptake inhibition American Journal of Psychiatry 2004 161 1256 1263 10.1176/appi.ajp.161.7.1256 15229059

[B29] Harmer CJ Mackay CE Reid CB Cowen PJ Goodwin GM Antidepressant drug treatment modifies the neural processing of nonconscious threat cues Biological Psychiatry 2006 59 816 820 10.1016/j.biopsych.2005.10.015 16460693

[B30] Harmer CJ O'Sullivan U Favaron E Massey-Chase R Ayres R Reinecke A Effect of Acute Antidepressant Administration on Negative Affective Bias in Depressed Patients American Journal of Psychiatry 2009 166 1178 1184 10.1176/appi.ajp.2009.09020149 19755572

[B31] Bhagwagar Z Cowen PJ Goodwin GM Harmer CJ Normalization of enhanced fear recognition by acute SSRI treatment in subjects with a previous history of depression American Journal of Psychiatry 2004 161 166 168 10.1176/appi.ajp.161.1.166 14702268

[B32] Sheline YI Barch DM Donnelly JM Ollinger JM Snyder AZ Mintun MA Increased amygdala response to masked emotional faces in depressed subjects resolves with antidepressant treatment: An fMRI study Biological Psychiatry 2001 50 651 658 10.1016/S0006-3223(01)01263-X 11704071

[B33] Miskowiak K O'Sullivan U Harmer CJ Erythropoietin reduces neural and cognitive processing of fear in human models of antidepressant drug action Biol Psychiatry 2007 62 1244 1250 10.1016/j.biopsych.2007.01.011 17553466

[B34] Miskowiak K O'Sullivan U Harmer CJ Erythropoietin enhances hippocampal response during memory retrieval in humans J Neurosci 2007 27 2788 2792 10.1523/JNEUROSCI.5013-06.2007 17360900 PMC6672568

[B35] Miskowiak K Inkster B O'Sullivan U Selvaraj S Goodwin GM Harmer CJ Differential effects of erythropoietin on neural and cognitive measures of executive function 3 and 7 days post-administration Exp Brain Res 2008 184 313 321 10.1007/s00221-007-1102-1 17828390

[B36] Miskowiak K Inkster B Selvaraj S Wise R Goodwin GM Harmer CJ Erythropoietin improves mood and modulates the cognitive and neural processing of emotion 3 days post administration Neuropsychopharmacology 2008 33 611 618 10.1038/sj.npp.1301439 17473836

[B37] Girgenti MJ Hunsberger J Duman CH Sathyanesan M Terwilliger R Newton SS Erythropoietin Induction by Electroconvulsive Seizure, Gene Regulation, and Antidepressant-Like Behavioral Effects Biological Psychiatry 2009 66 267 274 10.1016/j.biopsych.2008.12.005 19185286

[B38] Hariri AR Goldberg TE Mattay VS Kolachana BS Callicott JH Egan MF Brain-derived neurotrophic factor val66met polymorphism affects human memory-related hippocampal activity and predicts memory performance J Neurosci 2003 23 6690 6694 12890761 10.1523/JNEUROSCI.23-17-06690.2003 PMC6740735

[B39] Miskowiak KW Favaron E Hafizi S Inkster B Goodwin GM Cowen PJ Effects of erythropoietin on emotional processing biases in patients with major depression: an exploratory fMRI study Psychopharmacology (Berl) 2009 207 133 142 10.1007/s00213-009-1641-1 19705104

[B40] Young RC Biggs JT Ziegler VE Meyer DA A rating scale for mania: reliability, validity and sensitivity Br J Psychiatry 1978 133 429 435 10.1192/bjp.133.5.429 728692

[B41] Beck AT Ward CH Mendelson M Mock J Erbaugh J An inventory for measuring depression Arch Gen Psychiatry 1961 4 561 571 13688369 10.1001/archpsyc.1961.01710120031004

[B42] Fava M Iosifescu DV Pedrelli P Baer L Reliability and validity of the Massachusetts general hospital cognitive and physical functioning questionnaire Psychother Psychosom 2009 78 91 97 10.1159/000201934 19218827

[B43] EuroQol--a new facility for the measurement of health-related quality of life. The EuroQol Group Health Policy 1990 16 199 208 10.1016/0168-8510(90)90421-9 10109801

[B44] Wing JK Babor T Brugha T Burke J Cooper JE Giel R SCAN. Schedules for Clinical Assessment in Neuropsychiatry Arch Gen Psychiatry 1990 47 589 593 2190539 10.1001/archpsyc.1990.01810180089012

[B45] Hojman P Brolin C Gissel H Brandt C Zerahn B Pedersen BK Erythropoietin over-expression protects against diet-induced obesity in mice through increased fat oxidation in muscles PLoS One 2009 4 e5894 10.1371/journal.pone.0005894 19521513 PMC2690401

[B46] McIntyre RS Rasgon NL Kemp DE Nguyen HT Law CW Taylor VH Metabolic syndrome and major depressive disorder: co-occurrence and pathophysiologic overlap Curr Diab Rep 2009 9 51 59 10.1007/s11892-009-0010-0 19192425

[B47] Smith DJ Muir WJ Blackwood DHR Neurocognitive impairment in euthymic young adults with bipolar spectrum disorder and recurrent major depressive disorder Bipolar Disorders 2006 8 40 46 10.1111/j.1399-5618.2006.00275.x 16411979

[B48] Ekman P Friesen WV Pictures of facial affect 1976 Palo Alto: Consulting Psychologists Press

[B49] Beccari M Seizures in dialysis patients treated with recombinant erythropoietin. Review of the literature and guidelines for prevention Int J Artif Organs 1994 17 5 13 8188400

[B50] Locatelli F Del VL Pozzoni P Pure red-cell aplasia "epidemic"--mystery completely revealed? Perit Dial Int 2007 27 Suppl 2 S303 S307 17556324

[B51] McKoy JM Stonecash RE Cournoyer D Rossert J Nissenson AR Raisch DW Epoetin-associated pure red cell aplasia: past, present, and future considerations Transfusion 2008 48 1754 1762 10.1111/j.1537-2995.2008.01749.x 18482185 PMC2730535

[B52] Phrommintikul A Haas SJ Elsik M Krum H Mortality and target haemoglobin concentrations in anaemic patients with chronic kidney disease treated with erythropoietin: a meta-analysis Lancet 2007 369 381 388 10.1016/S0140-6736(07)60194-9 17276778

[B53] Ehrenreich H Weissenborn K Prange H Schneider D Weimar C Wartenberg K Recombinant human erythropoietin in the treatment of acute ischemic stroke Stroke 2009 40 e647 e656 10.1161/STROKEAHA.109.564872 19834012

[B54] Fava M Davidson KG Definition and epidemiology of treatment-resistant depression Psychiatr Clin North Am 1996 19 179 200 10.1016/S0193-953X(05)70283-5 8827185

[B55] Fava M Diagnosis and definition of treatment-resistant depression Biol Psychiatry 2003 53 649 659 10.1016/S0006-3223(03)00231-2 12706951

[B56] Posternak MA Zimmerman M Therapeutic effect of follow-up assessments on antidepressant and placebo response rates in antidepressant efficacy trials: meta-analysis Br J Psychiatry 2007 190 287 292 10.1192/bjp.bp.106.028555 17401033

[B57] Ferrario E Ferrari L Bidoli P De CD Del VM De DS Treatment of cancer-related anemia with epoetin alfa: a review Cancer Treat Rev 2004 30 563 575 10.1016/j.ctrv.2004.04.005 15325036

[B58] Erbayraktar S Grasso G Sfacteria A Xie QW Coleman T Kreilgaard M Asialoerythropoietin is a nonerythropoietic cytokine with broad neuroprotective activity in vivo Proc Natl Acad Sci USA 2003 100 6741 6746 10.1073/pnas.1031753100 12746497 PMC164517

[B59] Erbayraktar S de LN de LA Knisely JP Erbayraktar Z Yilmaz O Carbamylated erythropoietin reduces radiosurgically-induced brain injury Mol Med 2006 12 74 80 10.2119/2006-00042.Erbayraktar 16953562 PMC1578768

[B60] Leist M Ghezzi P Grasso G Bianchi R Villa P Fratelli M Derivatives of erythropoietin that are tissue protective but not erythropoietic Science 2004 305 239 242 10.1126/science.1098313 15247477

[B61] King VR Averill SA Hewazy D Priestley JV Torup L Michael-Titus AT Erythropoietin and carbamylated erythropoietin are neuroprotective following spinal cord hemisection in the rat Eur J Neurosci 2007 26 90 100 10.1111/j.1460-9568.2007.05635.x 17614942

[B62] Mahmood A Lu D Qu C Goussev A Zhang ZG Lu C Treatment of traumatic brain injury in rats with erythropoietin and carbamylated erythropoietin J Neurosurg 2007 107 392 397 10.3171/JNS-07/08/0392 17695395

[B63] Wang Y Zhang ZG Rhodes K Renzi M Zhang RL Kapke A Post-ischemic treatment with erythropoietin or carbamylated erythropoietin reduces infarction and improves neurological outcome in a rat model of focal cerebral ischemia Br J Pharmacol 2007 151 1377 1384 10.1038/sj.bjp.0707285 17603558 PMC2189829

[B64] Yu YP Xu QQ Zhang Q Zhang WP Zhang LH Wei EQ Intranasal recombinant human erythropoietin protects rats against focal cerebral ischemia Neurosci Lett 2005 387 5 10 10.1016/j.neulet.2005.07.008 16054296

